# LncRNA HOTAIR facilitates high glucose-induced mesangial cell proliferation, fibrosis and oxidative stress in diabetic nephropathy via regulating miR-147a/WNT2B axis

**DOI:** 10.1186/s13098-022-00802-3

**Published:** 2022-02-22

**Authors:** Xin Wang, Wei Wang, Weizhong HuangFu, Zhonghua Liu, Feng Zhao

**Affiliations:** 1 Department of General, Affiliated Hospital of Inner Mongolia Medical University, Mengrong Central Home, Donghongqiao Street, Hohhot, 010010 China; 2 Department of Orthopaedics, Inner Mongolia People’s Hospital, Hohhot, 010010 China

**Keywords:** Diabetic nephropathy, lncRNA HOTAIR, miR-147a, WNT2B

## Abstract

**Background:**

Long non-coding RNAs (lncRNAs) have been shown to be involved in the regulation of many disease progression. However, the role of lncRNA HOX transcript antisense RNA (HOTAIR) in diabetic nephropathy (DN) remains unclear.

**Methods:**

High glucose (HG)-induced human mesangial cells (HMC) was used to construct DN cell models in vitro. HMC proliferation was evaluated by CCK8 assay and EDU staining. Protein levels of proliferation markers, fibrosis markers, and wingless-type family member 2B (WNT2B) were measured using western blot analysis. HMC oxidative stress was assessed by determining the levels of oxygen species and malondialdehyde, as well as superoxide dismutase activity. Relative expression levels of lncRNA HOTAIR, microRNA (miR)-147a, and WNT2B were examined using quantitative real-time PCR. The interaction between miR-147a and lncRNA HOTAIR or WNT2B was confirmed by dual-luciferase reporter assay and RIP assay.

**Results:**

Our data showed that lncRNA HOTAIR knockdown could inhibit the proliferation, fibrosis, and oxidative stress in HG-induced HMC. LncRNA HOTAIR could serve as a sponge of miR-147a. The inhibition effect of lncRNA HOTAIR silencing on the biological functions of HG-induced HMC could be reversed by miR-147a inhibitor. WNT2B was targeted by miR-147a, and its overexpression also overturned the suppressive effect of miR-147a on the proliferation, fibrosis, and oxidative stress of HG-induced HMC.

**Conclusion:**

In total, our research pointed out that lncRNA HOTAIR could mediate miR-147a/WNT2B axis to promote DN progression.

**Supplementary Information:**

The online version contains supplementary material available at 10.1186/s13098-022-00802-3.

## Background

Diabetic nephropathy (DN) is a common microvascular complication of diabetes, which is generally related to metabolic abnormalities caused by hyperglycemia [1, 2]. Current research believes that renal interstitial fibrosis, hyperproliferation and oxidative stress are one of the main reasons for the development of DN [3, 4]. Therefore, clarifying the mechanism of DN progression and finding effective therapeutic targets have become important topics in the study of DN. At present, human mesangial cells (HMC) induced by high glucose (HG) are often used to construct in vitro cell models of DN [5, 6].

Long noncoding RNA (lncRNA) is a type of RNA transcript that does not encode protein, and its abnormal expression has been confirmed to be related to human diseases progression [7, 8]. LncRNA can be used as a competitive endogenous RNA (ceRNA) to regulate the aggregation and functions of microRNA (miRNA), which indirectly affects the expression of target mRNAs [9, 10]. More and more studies have confirmed that lncRNA is closely related to the occurrence and progression of DN [11]. For example, lncRNA GAS5 was confirmed to hinder mesangial cell proliferation and fibrosis through the miR-221/SIRT1 axis, so it might play a negative role in DN progression [12]. On the contrary, high expression of lncRNA KCNQ1OT1 in DN could accelerate HG-induced HMC proliferation, oxidative stress and extracellular matrix (ECM) deposition, thus promoting the development of DN [13]. All these evidences make us believe that lncRNAs are an important participants in DN development.

HOX transcript antisense RNA (HOTAIR) is the first lncRNA that has been found to have trans-transcriptional regulation. In many previous studies, lncRNA HOTAIR has been proved to be a pro-cancer factor to mediate cancer malignant progression, including breast cancer [14], hepatocellular carcinoma [15] and gastric cancer [16]. Studies had pointed out that the expression of lncRNA HOTAIR was increased in human diabetic kidney disease and HG-induced podocytes [17], but the current research on the role of HOTAIR in DN has not been reported. In this study, the function of lncRNA HOTAIR in DN was determined by exploring the effect of lncRNA HOTAIR on the biological functions of HMC under HG environment. Through the lncRNA/miRNA/mRNA network, our research explored the molecular mechanism of lncRNA HOTAIR to provide a new perspective for further understanding of the pathogenesis of DN.

## Materials and methods

### Cell culture, treatment and transfection

HMC (BeNa Culture Collection, Beijing, China) was cultured in normal glucose (NG, 5 mM) DMEM medium (Gibco, Grand Island, NY, USA) supplemented with 10% FBS (Gibco) and 1% penicillin/streptomycin (Invitrogen, Carlsbad, CA, USA) at 37 ℃ with 5% CO_2_. For constructing DN cell model, HMC was cultured in DMEM medium containing HG (25 mM, Sigma-Aldrich, St. Louis, MO, USA) for 48 h. For cell transfection, HMC was seeded into 6-well plates and serum starved (0.2% FBS) overnight. The small interference RNA of lncRNA HOTAIR (si-lncRNA HOTAIR), miR-147a mimic or inhibitor, pcDNA wingless-type family member 2B (WNT2B) overexpression vector (pcDNA-WNT2B) or their negative controls (si-NC, mimic NC, inhibitor NC, or pcDNA) were synthesized from RiboBio (Guangzhou, China). Lipofectamine 3000 (Invitrogen) was used to transfect them into HMC after the cell density reached 60%.

### Cell counting kit 8 (CCK8) assay

HMC was seeded in 96-well plate (2 × 10^4^ cells/well) and cultured overnight. At the specified point in time, each well was exposed to 10 μL CCK8 reagent (Dojindo, Kumamoto, Japan) for 4 h. The optical density (OD) value was determined by a microplate reader.

### EDU staining

HMC was seeded in 24-well plates and cultured for 24 h. In accordance with the instructions of EDU Cell Proliferation Kit (Beyotime, Shanghai, China), the cells were incubated with EDU and stained with DAPI for cell nuclei. Fluorescence images were captured under a fluorescence microscope and the percentage of EDU positive cells was counted.

### Western blot (WB) analysis

The proteins were lysed by RIPA lysate buffer (Elabscience, Wuhan, China) and its concentration was quantified using BCA Protein Concentration Kit (Elabscience). The protein (20 μg) was isolated by SDS-PAGE gel and transferred to PVDF membrane (Roche, Basel, Switzerland) followed by hatched with 5% skim milk. The membrane was incubated with various primary antibodies and labeled with secondary antibody. The protein blots were visualized with Western Chemiluminescent HRP Substrate (Millipore, Billerica, MA, USA). The antibodies used in this study including anti-PCNA (1:1000, ab18197), anti-CyclinD1 (1:5000, ab226977), anti-fibronectin (1:1000, ab2413), anti-collagen I (1:2000, ab34710), anti-α-SMA (1:1000, ab5694), anti-WNT2B (1:200, ab203225), anti-β-actin (1: 1000, ab5694) and Goat Anti-Rabbit IgG (1:50,000, ab205718), which were bought from Abcam (Cambridge, MA, USA).

### Detection of oxidative stress

According to the kit instructions, reactive oxygen species (ROS) level was detected using Cellular ROS Assay Kit (Abcam). Meanwhile, malondialdehyde (MDA) level and superoxide dismutase (SOD) activity were measured in the cellular supernatant using MDA and SOD Assay Kits (Sigma-Aldrich).

### Quantitative real-time PCR (qRT-PCR)

Total RNA was extracted using Trizol reagent (Invitrogen). The cDNA was obtained using TaqMan Reverse Transcription Reagent (Invitrogen) or miScript II RT Kit (Qiagen, Duesseldorf, Germany). QRT-PCR analysis was conducted on PCR system by means of the SYBR Green (Takara, Dalian, China). The 2^−ΔΔCt^ method was utilized to calculate the relative expression with β-actin or U6 as internal control. Primers were shown as below: lncRNA HOTAIR, F 5'-TTGCCCCCAGCAAGAATCAT-3', R 5'-GTTCCGGAAATCAGGGCAGA-3'; miR-147a, F 5'-GCCGAGGTGTGTGGAAATGC-3', R 5'-CAGTGCGTGTCGTGGAGT-3'; WNT2B, F 5'-GGGGCACGAGTGATCTGTG-3', R 5'-GCATGATGTCTGGGTAACGCT-3'; β-actin, F 5'-CTCCATCCTGGCCTCGCTGT-3', R 5'-GCTGTCACCTTCACCGTTCC-3'; U6, F 5'-CTCGCTTCGGCAGCACATA-3', R 5'-CGAATTTGCGTGTCATCCT-3'.

### Dual-luciferase reporter assay

The wild-type (wt) or mutant-type (mut) reporter vectors of lncRNA HOTAIR and WNT2B (lncRNA HOTAIR wt/mut and WNT2B 3'UTR wt/mut) were constructed via cloning their binding sequences or mutate sequences with miR-147a into psiCHECK-2 reporter vectors. 293T cells were co-transfected with these generated vectors and miR-147a mimic or mimic NC for 48 h. Then, the luciferase activity was determined with a Dual-luciferase Analysis Kit (Beyotime).

### RIP assay

Basing on the instructions of Magna RIP Kit (Millipore), the lysates of HMC were incubated with anti-Ago2/anti-IgG-conjugated magnetic beads for 4 h. The enrichment of lncRNA HOTAIR and miR-147a in the immunoprecipitated RNA were measured via qRT-PCR.

### Statistical analysis

Statistical analysis was performed using GraphPad Prism 7.0 (GraphPad, La Jolla, CA, USA). All data were calculated as mean ± standard deviation. Student’s *t*-test or one way analysis of variance was performed to compare the statistical differences between groups. *P* < 0.05 was considered significant.

## Results

### HG induced HMC proliferation, fibrosis and oxidative stress

**Fig. 1:**
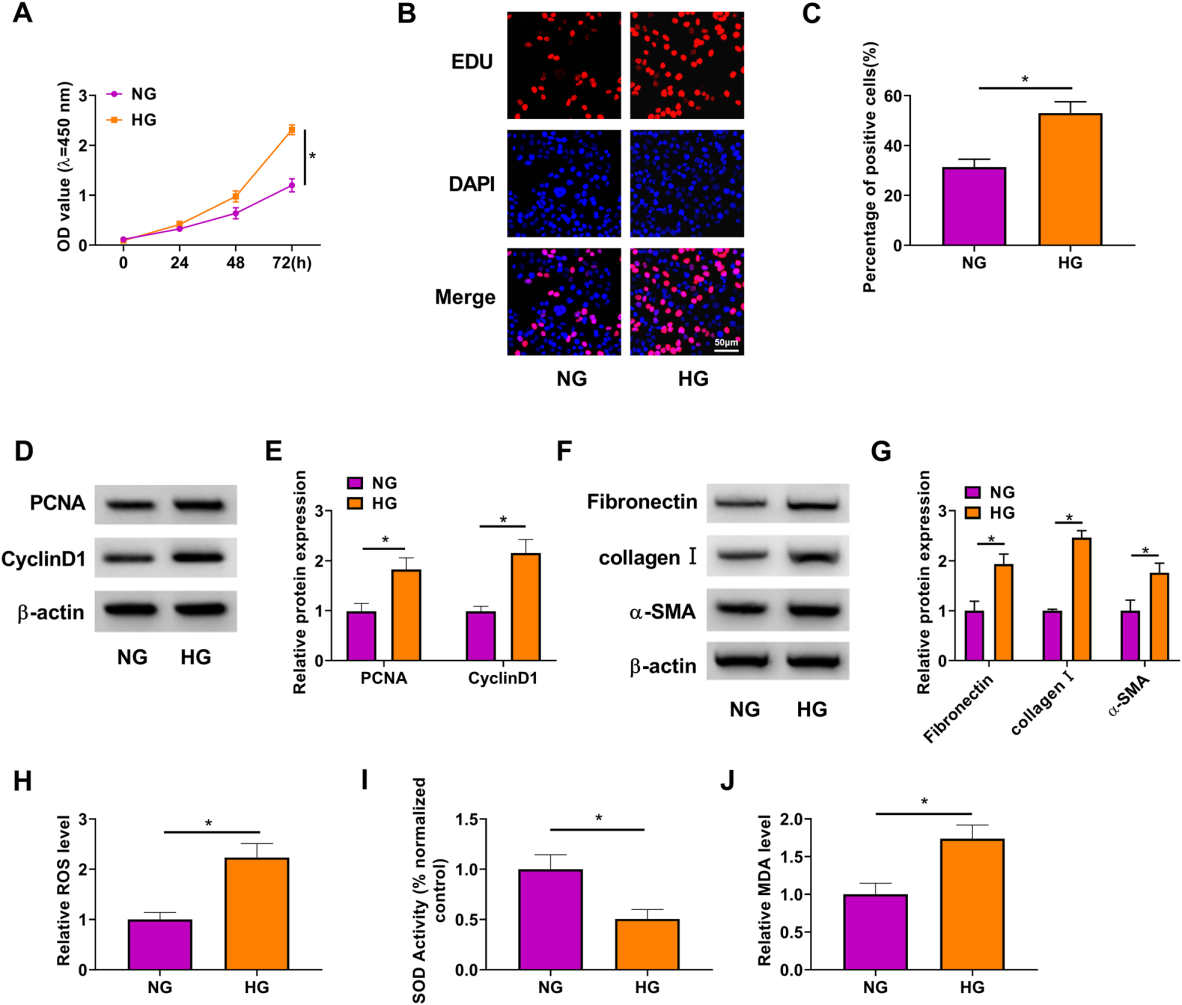
HG induced HMC proliferation, fibrosis and oxidative stress. HMC was cultured under HG treatment or NG treatment. **A** CCK8 assay was used to measure HMC viability. **B**, **C** EDU staining was performed to evaluate the percentage of EDU positive cells. **D**–**G** The protein levels of PCNA, CyclinD1, fibronectin, collagen I and α-SMA were determined by WB analysis. **H**–**J** The ROS level, SOD activity and MDA level were examined by corresponding Assay Kits. *P < 0.05

To determine whether HG-induced DN cell model in vitro was successful, we evaluated the proliferation, fibrosis, and oxidative stress capacity of HMC. Our data showed that HG treatment could not only promote the viability of HMC (Fig. 1A), but also increase the percentage of EDU positive cells (Fig. 1B, C). Besides, the protein levels of proliferation markers PCNA and CyclinD1 also were enhanced in HMC after HG treatment (Fig. 1D, E). By measuring fibrosis markers expression, we confirmed that HG treatment increased the protein expression of fibronectin, collagen I and α-SMA in HMC (Fig. 1F, G). In addition, the levels of ROS and MDA were markedly raised, while SOD activity was significantly decreased in HMC treated with HG (Fig. 1H–J). These data suggested that HG treatment could be used to induce DN cell model in vitro.

### Knockdown of lncRNA HOTAIR inhibited the biological functions of HG-induced HMC

**Fig. 2:**
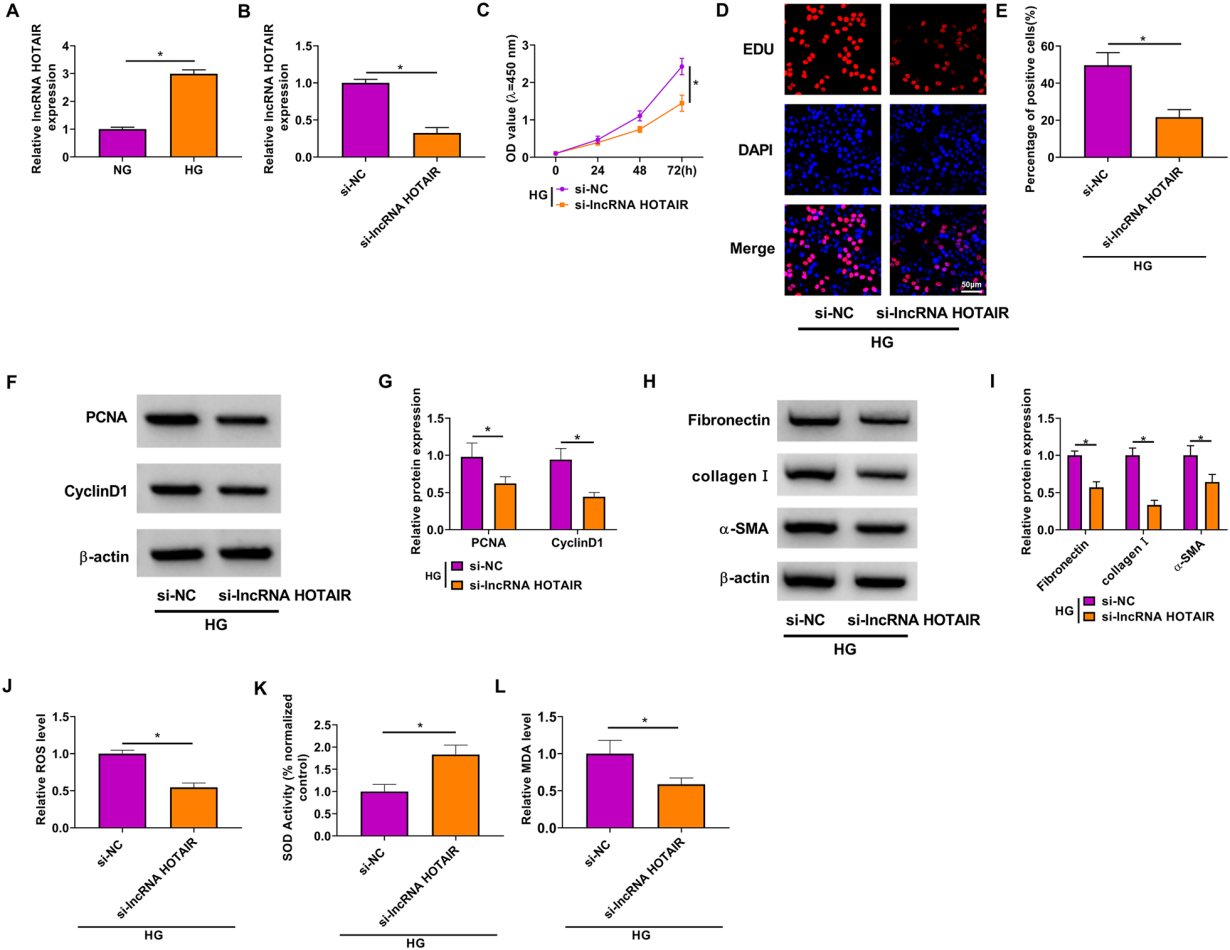
Effects of lncRNA HOTAIR silencing on the biological functions of HG-induced HMC. **A** LncRNA HOTAIR expression was detected by qRT-PCR in HMC treated with HG or NG. **B** QRT-PCR was used to measure lncRNA HOTAIR expression in HMC transfected with si-NC or sh-lncRNA HOTAIR. **C**–**L** Transfected HMC was treated with HG. **C** HMC viability was assessed by CCK8 assay. **D**–**E** The percentage of EDU positive cells was evaluated by EDU staining. **F**–**I** The protein levels of PCNA, CyclinD1, fibronectin, collagen I and α-SMA were examined using WB analysis. **J**–**L** Corresponding Assay Kits were used to determine the ROS level, SOD activity and MDA level. *P < 0.05

In HG-induced HMC, lncRNA HOTAIR was markedly enhanced compared with that in HMC treated with NG (Fig. 2A). To determine the role of lncRNA HOTAIR in DN progression, HMC was transfected with the siRNA of lncRNA HOTAIR. As exhibited in Fig. 2B, the expression of lncRNA HOTAIR was significantly reduced after transfection. Then, the transfected HMC was treated with HG. Compared to the control group, lncRNA HOTAIR silencing remarkably inhibited the viability of HMC and the percentage of EDU positive cells (Fig. 2C, E). Also, the protein levels of PCNA, CyclinD1, fibronectin, collagen I and α-SMA also were decreased in HG-induced HMC treated with si-lncRNA HOTAIR (Fig. 2F, I). Furthermore, lncRNA HOTAIR knockdown also suppressed ROS and MDA levels, while enhanced SOD activity in HG-induced HMC (Fig. 2J–L). In addition, we assessed cell apoptosis after lncRNA HOTAIR knockdown, and confirmed that lncRNA HOTAIR knockdown enhanced the apoptosis of HG-induced HMC (Additional file 1: Figure S1). Therefore, we proposed that lncRNA HOTAIR might promote DN progression.

### LncRNA HOTAIR could act as a sponge of miR-147a

**Fig. 3:**
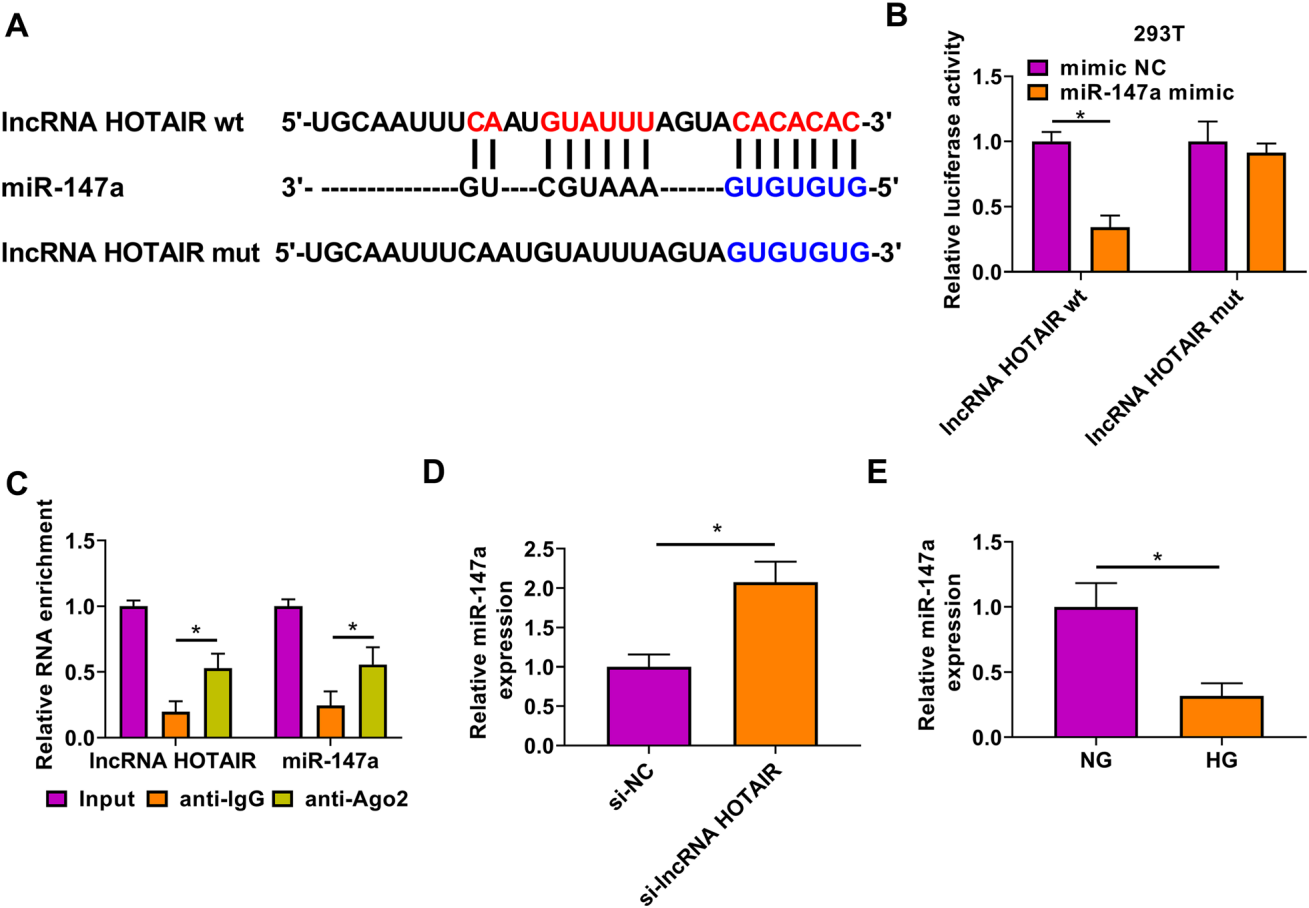
LncRNA HOTAIR could act as a sponge of miR-147a. **A** The sequences of lncRNA HOTAIR wt/mut were shown. Dual-luciferase reporter assay (**B**) and RIP assay (**C**) were used to assess the interaction between lncRNA HOTAIR and miR-147a. **D** MiR-147a expression was measured by qRT-PCR in HMC transfected with si-NC or si-lncRNA HOTAIR. **E** The expression of miR-147a was detected by qRT-PCR in HMC treated with HG or NG. *P < 0.05

The Lncbase2.0 software (http://carolina.imis.athena-innovation.gr/diana_tools/web/index.php?r=lncbasev2/index) was used to predict the targeted miRNA for lncRNA HOTAIR. As a result, miR-147a was found to have complementary binding sites with lncRNA HOTAIR (Fig. 3A). Dual-luciferase reporter assay results suggested that miR-147a mimic reduced the luciferase activity of lncRNA HOTAIR wt vector, while had not effect on that of the lncRNA HOTAIR mut vector (Fig. 3B). Also, lncRNA HOTAIR and miR-147a were discovered to be markedly enriched in anti-Ago2 compared to anti-IgG (Fig. 3C). In HMC transfected with si-lncRNA HOTAIR, miR-147a expression was significantly enhanced compared to the control group (Fig. 3D). Under the treatment of HG, miR-147a expression was decreased in HMG (Fig. 3E), which was contrary to the expression trend of lncRNA HOTAIR. These results showed that miR-147a could be sponged by lncRNA HOTAIR.

### MiR-147a inhibitor reversed the regulation of lncRNA HOTAIR silencing on the biological functions of HG-induced HMC

**Fig. 4:**
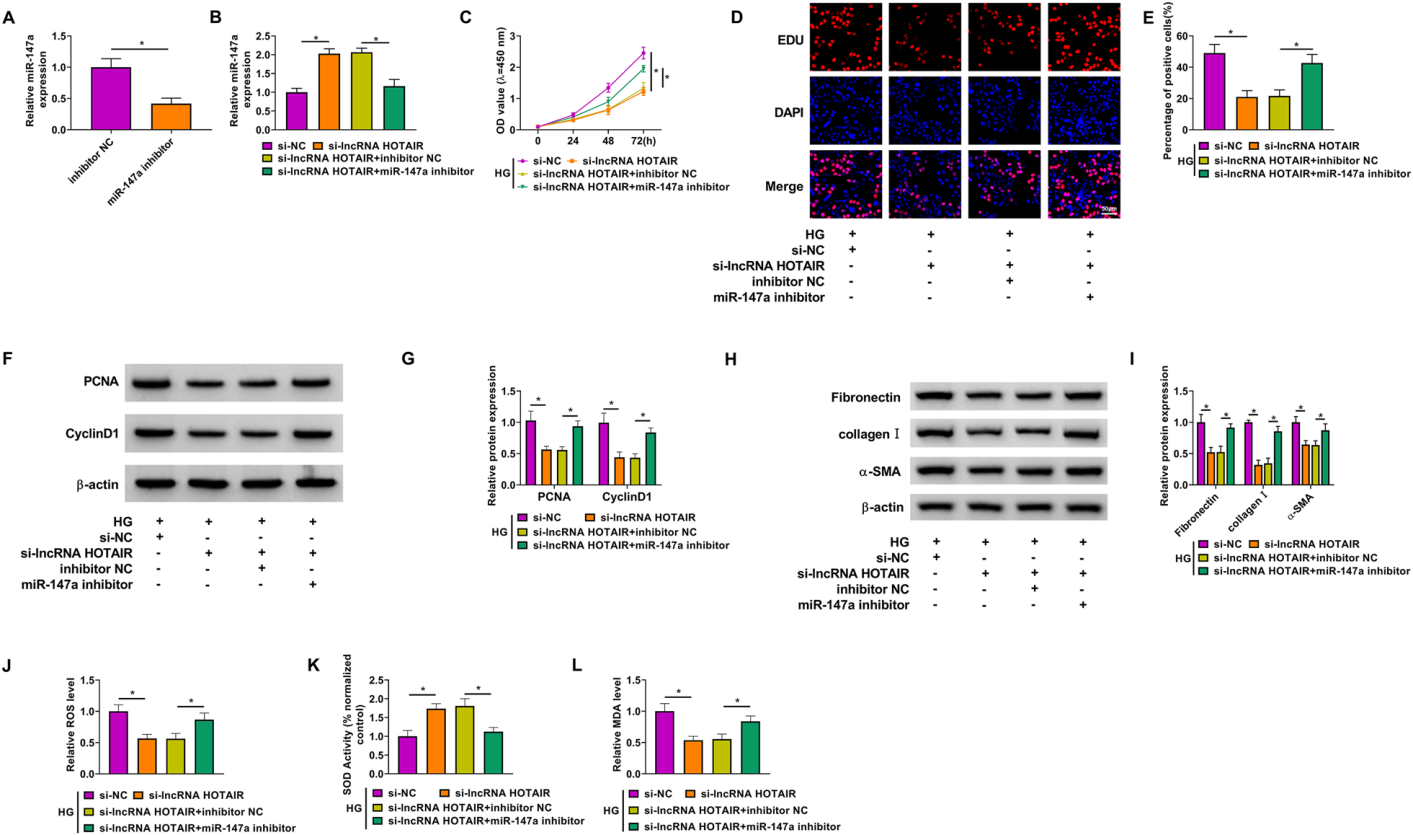
Effects of lncRNA HOTAIR silencing and miR-147a inhibitor on the biological functions of HG-induced HMC. **A** The transfection efficiency of miR-147a inhibitor was confirmed by detecting miR-147a expression using qRT-PCR. **B** HMC was transfected with si-NC, si-lncRNA HOTAIR, si-lncRNA HOTAIR + inhibitor NC or si-lncRNA HOTAIR + miR-147a inhibitor. The expression of miR-147a was measured by qRT-PCR. **C**–**L** Transfected HMC was treated with HG. **C** CCK8 assay was performed to detect HMC viability. **D**–**E** EDU staining was used to examine the percentage of EDU positive cells. **F**–**I** WB analysis was utilized for measuring the protein levels of PCNA, CyclinD1, fibronectin, collagen I and α-SMA. **J**–**L** The ROS level, SOD activity and MDA level were analyzed using corresponding Assay Kits. *P < 0.05

To further explore whether lncRNA HOTAIR regulated DN progression by sponging miR-147a, we performed the rescue experiments. After confirming that miR-147a inhibitor indeed reduced miR-147a expression in HMC (Fig. 4A), we co-transfected with si-lncRNA HOTAIR and miR-147a inhibitor into HMC. The detection results of miR-147a expression showed that the addition of miR-147a inhibitor could decrease miR-147a expression promoted by lncRNA HOTAIR silencing (Fig. 4B). After the transfected HMC was treated with HG, we discovered that the suppressive effect of lncRNA HOTAIR knockdown on HMC viability and the percentage of EDU positive cells could be reversed by miR-147a inhibitor (Fig. 4C, E). Moreover, inhibition of miR-147a also abolished the reduction effect of lncRNA HOTAIR downregulation on the protein levels of PCNA, CyclinD1, fibronectin, collagen I and α-SMA in HG-induced HMC (Fig. 4F–I). Meanwhile, the decreasing effect of lncRNA HOTAIR knockdown on the levels of ROS and MDA, and the increasing effect on SOD activity in HG-induced HMC also were overturned by miR-147a inhibitor (Fig. 4J–L). All data revealed that lncRNA HOTAIR sponged miR-147a to mediate DN progression.

### MiR-147a targeted WNT2B

**Fig. 5:**
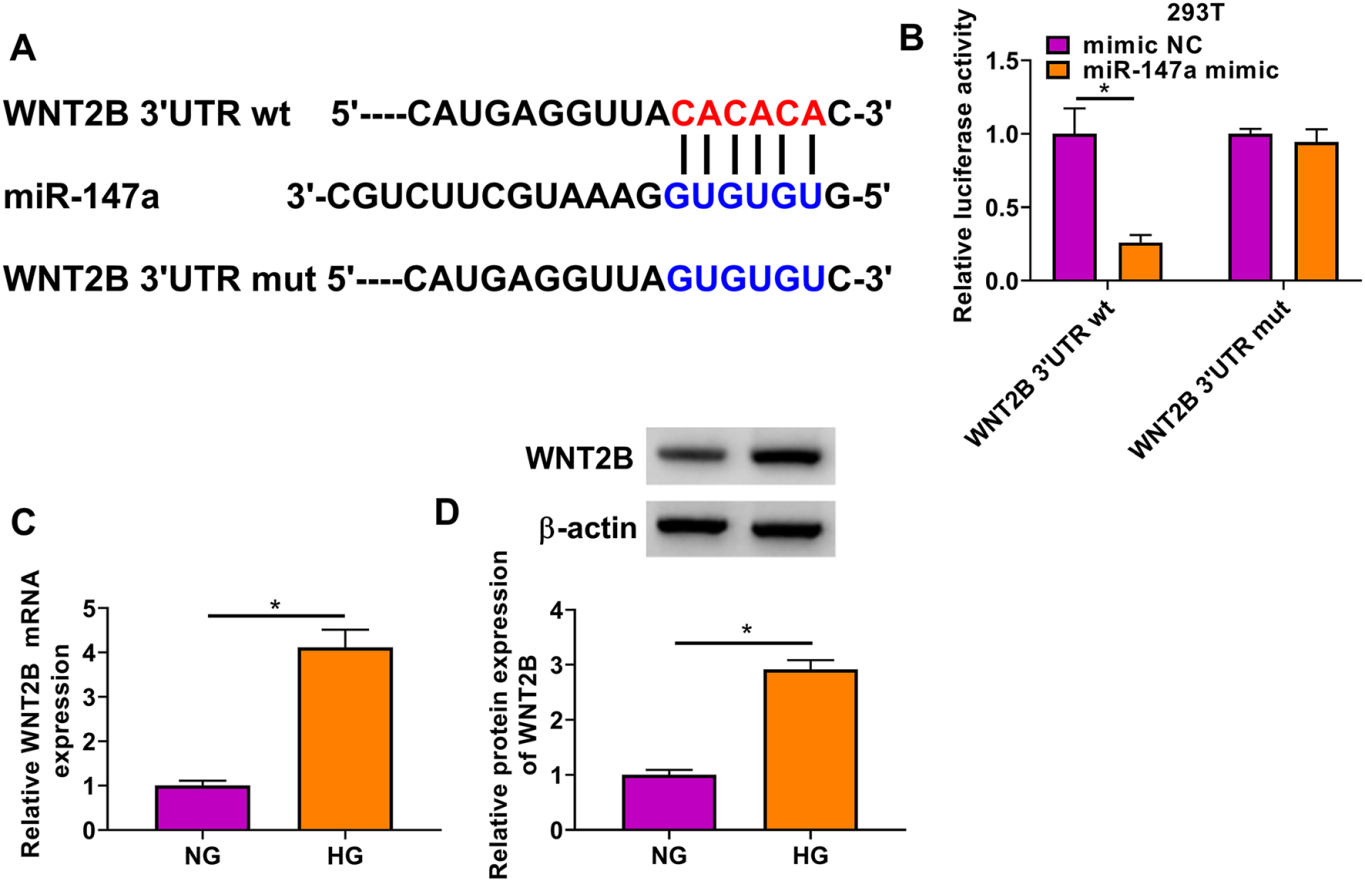
MiR-147a targeted WNT2B. **A** The sequences of WNT2B 3'UTR wt/mut were presented. **B** Dual-luciferase reporter assay was utilized for evaluating the interaction between WNT2B and miR-147a. **C**–**D** The mRNA and protein expression of WNT2B was determined using qRT-PCR and WB analysis in HMC treated with HG or NG. *P < 0.05

To confirm the target of miR-147a, the Starbase3.0 software (http://starbase.sysu.edu.cn/) was used and the WNT2B 3'UTR was found to can bound with miR-147a (Fig. 5A). The results of dual-luciferase reporter assay suggested that the luciferase activity of WNT2B 3'UTR wt vector could be inhibited by miR-147a mimic, while the luciferase activity of WNT2B 3'UTR mut vector was not affected by miR-147a mimic (Fig. 5B). In addition, we discovered that WNT3B expression was significantly upregulated in HG-induced HMC at the mRNA level and protein level (Fig. 5C–D). The above data showed that WNT2B might be a target of miR-147a.

### Overexpression of WNT2B reversed the regulation of miR-147a on the proliferation, fibrosis and oxidative stress of HG-induced HMC

**Fig. 6:**
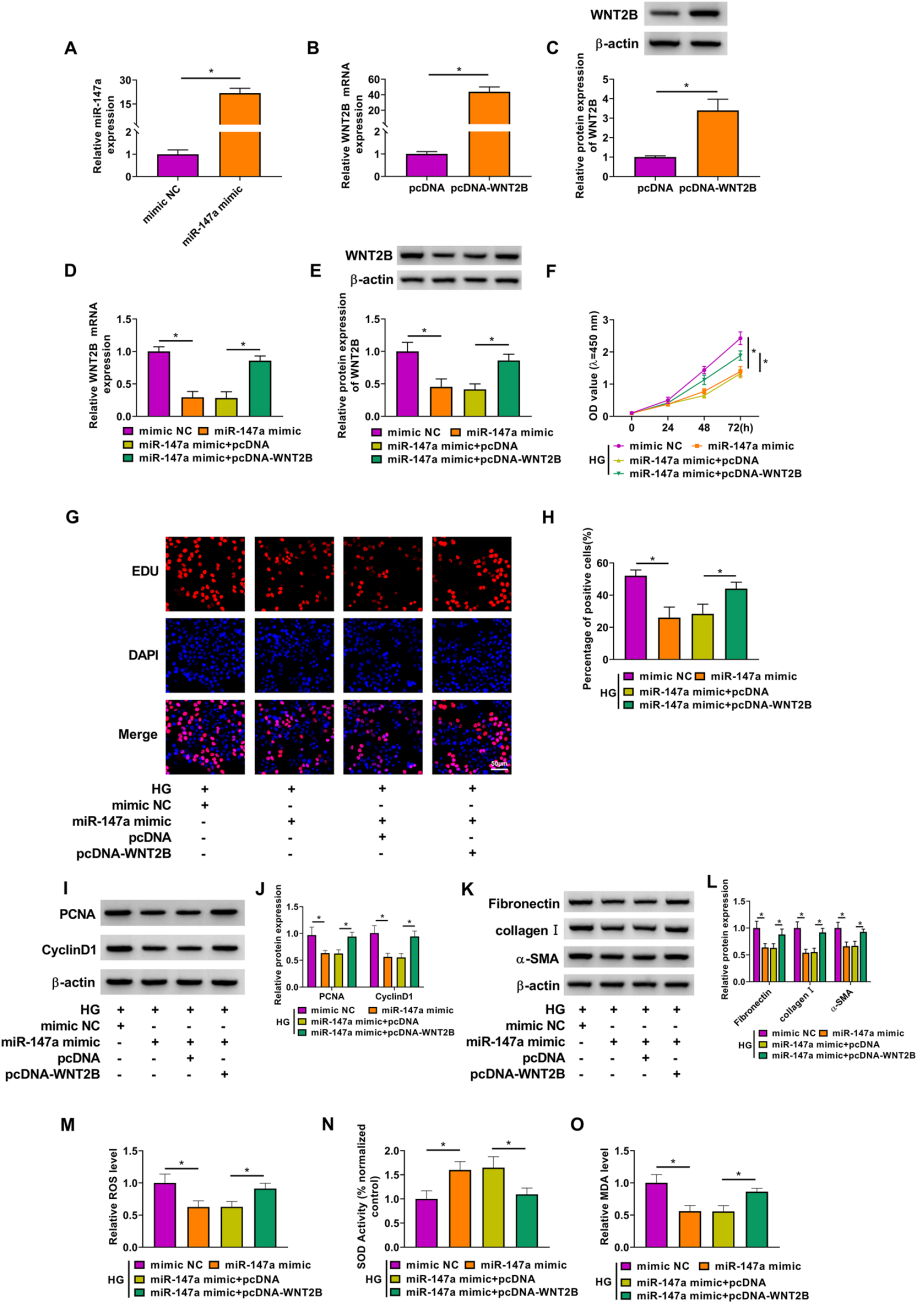
Effects of miR-147a and WNT2B on the proliferation, fibrosis and oxidative stress of HG-induced HMC. **A** The transfection efficiency of miR-147a mimic was determined by measuring miR-147a expression using qRT-PCR. **B**–**C** QRT-PCR and WB analysis were used to detect WNT2B mRNA and protein expression to evaluate the transfection efficiency of pcDNA-WNT2B. **D**–**E** HMC was transfected with mimic NC, miR-147a mimic, miR-147a mimic + pcDNA or miR-147a mimic + pcDNA-WNT2B. The mRNA and protein expression of WNT2B was examined by qRT-PCR and WB analysis. **F**–**O** Transfected HMC was treated with HG. **F** CCK8 assay was employed to test HMC viability. **G**–**H** The percentage of EDU positive cells was assessed using EDU staining. **I**–**L** WB analysis was performed to detect he protein levels of PCNA, CyclinD1, fibronectin, collagen I and α-SMA. **M**–**O** Corresponding Assay Kits were utilized to assess the ROS level, SOD activity and MDA level. *P < 0.05

**Fig. 7:**
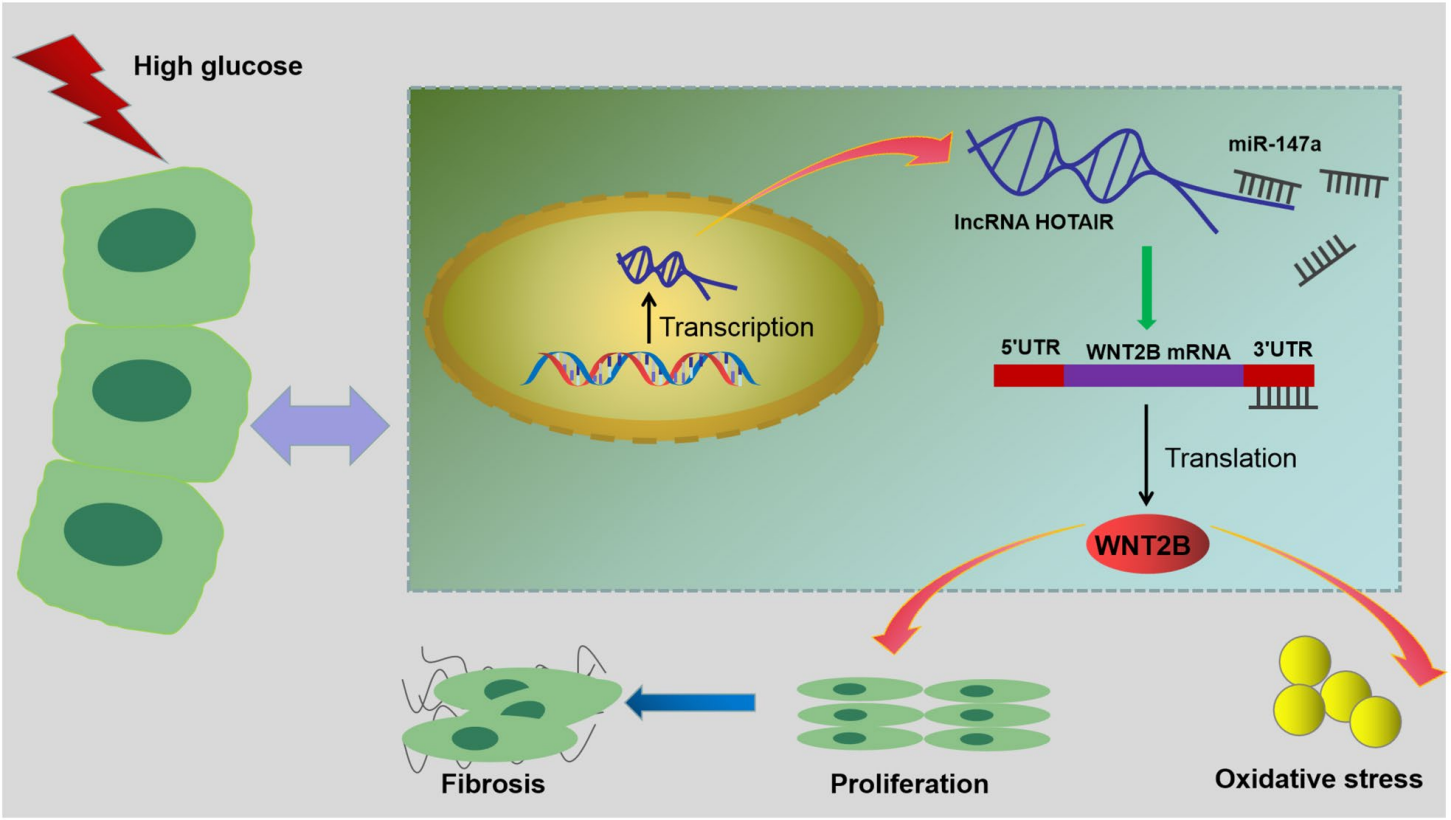
Summary diagram of this study. Under HG condition, lncRNA HOTAIR could regulate the miR-147a/WNT2B axis, thereby promoting the proliferation, fibrosis and oxidative stress of HMC

Then, the rescue experiments were performed to explore whether miR-147a regulated DN progression via targeting WNT2B. Firstly, we confirmed that miR-147a mimic could promote miR-147a expression, and pcDNA-WNT2B also could enhance the mRNA and protein expression of WNT2B in HMC (Fig. 6A–C). After that, miR-147a mimic and pcDNA-WNT2B were co-transfected into HMC. As shown in Fig. 6D–E, miR-147a mimic could inhibit the mRNA and protein expression of WNT2B, while this effect could be abolished by pcDNA-WNT2B (Fig. 6D–E). After the HMC was treated with HG, we found that miR-147a overexpression could repress the viability and reduce the percentage of EDU positive cells, and these effects could be reversed by overexpressing WNT2B (Fig. 6F–H). Also, the protein levels of PCNA, CyclinD1, fibronectin, collagen I and α-SMA in HG-induced HMC suppressed by miR-147a mimic also could be overturned by WNT2B overexpression (Fig. 6I, L). Additionally, miR-147a mimic reduced the levels of ROS and MDA, while accelerated the activity of SOD in HG-induced HMC. However, these effects also could be reversed by WNT2B upregulation (Fig. 6M–O). Hence, we confirmed that miR-147a targeted WNT2B to inhibit DN progression. Above all, our results proposed that lncRNA HOTAIR promoted the proliferation, fibrosis and oxidative stress of HG-induced HMC to aggravate DN progression by regulating the miR-147a/WNT2B axis (Fig. 7).

## Discussion

Current research believes that hyperglycemia is the primary cause of DN, which can promote cell proliferation, oxidative stress and fibrosis, and ultimately lead to the initiation and development of DN [18, 19]. A large number of lncRNA has been confirmed to be a potential target for DN therapy [20, 21]. In previous studies, Zhang et al*.* suggested that lncRNA HOTAIR could alleviate the occurrence of rheumatoid arthritis by promoting chondrocytes proliferation and inhibiting inflammatory response [22]. Zhan et al*.* proposed that lncRNA HOTAIR knockdown might relieve intervertebral disc degenerative changes by suppressing nucleus pulposus cell senescence, apoptosis, and ECM degradation [23]. Here, our data showed that lncRNA HOTAIR was significantly upregulated in HG-induced HMC, which was consistent with the past research [17]. In HG-promoted HMC proliferation, fibrosis and oxidative stress, we found that lncRNA HOTAIR silencing could alleviate these process. These evidences suggested that knockdown of lncRNA HOTAIR might be an effective way to treat DN.

Bioinformatics software analysis showed that miR-147a could be targeted by lncRNA HOTAIR. In cancer-related studies, miR-147a has been identified as a tumor suppressor to regulate cancer progression, such as non-small-cell lung cancer [24] and cervical cancer [25]. MiR-147a might inhibit cellular inflammation to mitigate pneumonia process [26]. Ji et al*.* reported that miR-147a was downregulated in DN patients and HG-induced HMC and could repress HG-induced HMC proliferation and fibrosis [27]. Consistent with the results of previous studies, we also showed that miR-147a had suppressive effects on the proliferation and fibrosis of HG-induced HMC, and could hinder oxidative stress. Moreover, our study indicated that miR-147a inhibitor reversed the inhibitory effect of lncRNA HOTAIR knockdown on cell proliferation, fibrosis and oxidative stress, further confirming that lncRNA HOTAIR indeed mediated the biological functions of HG-induced HMC through targeting miR-147a.

WNT2B is a key inducer of WNT signal transduction, and its abnormal expression is associated with the occurrence and development of human cancers [28, 29]. Notably, the role of WNT2B in the progression of DN has been discovered. Zhu et al*.* showed that WNT2B was upregulated in DN mice and HG-induced mesangial cells, which might accelerate DN progression by enhancing cell proliferation, inflammation, and ECM accumulation [30]. Also, lncRNA CDKN2B increased WNT2B expression to promote HG-induced HMC proliferation and ECM accumulation via sponging miR-15b-5p [31]. In this, we confirmed that WNT2B could be targeted by miR-147a, and its expression was positively regulated by lncRNA HOTAIR. The reversal effect of WNT2B on miR-147a-mediated cell functions revealed that miR-147a targeted WNT2B to suppress HG-induced proliferation, fibrosis and oxidative stress in HMC.

## Conclusions

To sum up, our research showed that lncRNA HOTAIR facilitated HG-induced HMC proliferation, fibrosis and oxidative stress through the miR-147a/WNT2B regulatory pathway. Our research revealed the role of lncRNA HOTAIR in DN progression, suggesting that lncRNA HOTAIR might be a potential target for the treatment of DN.

## Supplementary Information

**Additional file 1: Figure S1.** Effects of lncRNA HOTAIR silencing on the apoptosis of HG-induced HMC. HMC was transfected with si-NC or si-lncRNA HOTAIR, and then treated with HG. The apoptosis rate of HMC was assessed by flow cytometry. *P < 0.05.

## Data Availability

The data sets used and/or analyzed during the current study are available from the corresponding author on reasonable request.
