# Supplementary figures and images for: LncRNA HOTAIR facilitates high glucose-induced mesangial cell proliferation, fibrosis and oxidative stress in diabetic nephropathy via regulating miR-147a/WNT2B axis
Source: Diabetol Metab Syndr. 2022 Feb 22;14:33. doi: 10.1186/s13098-022-00802-3 (PMC8864868; doi:10.1186/s13098-022-00802-3)

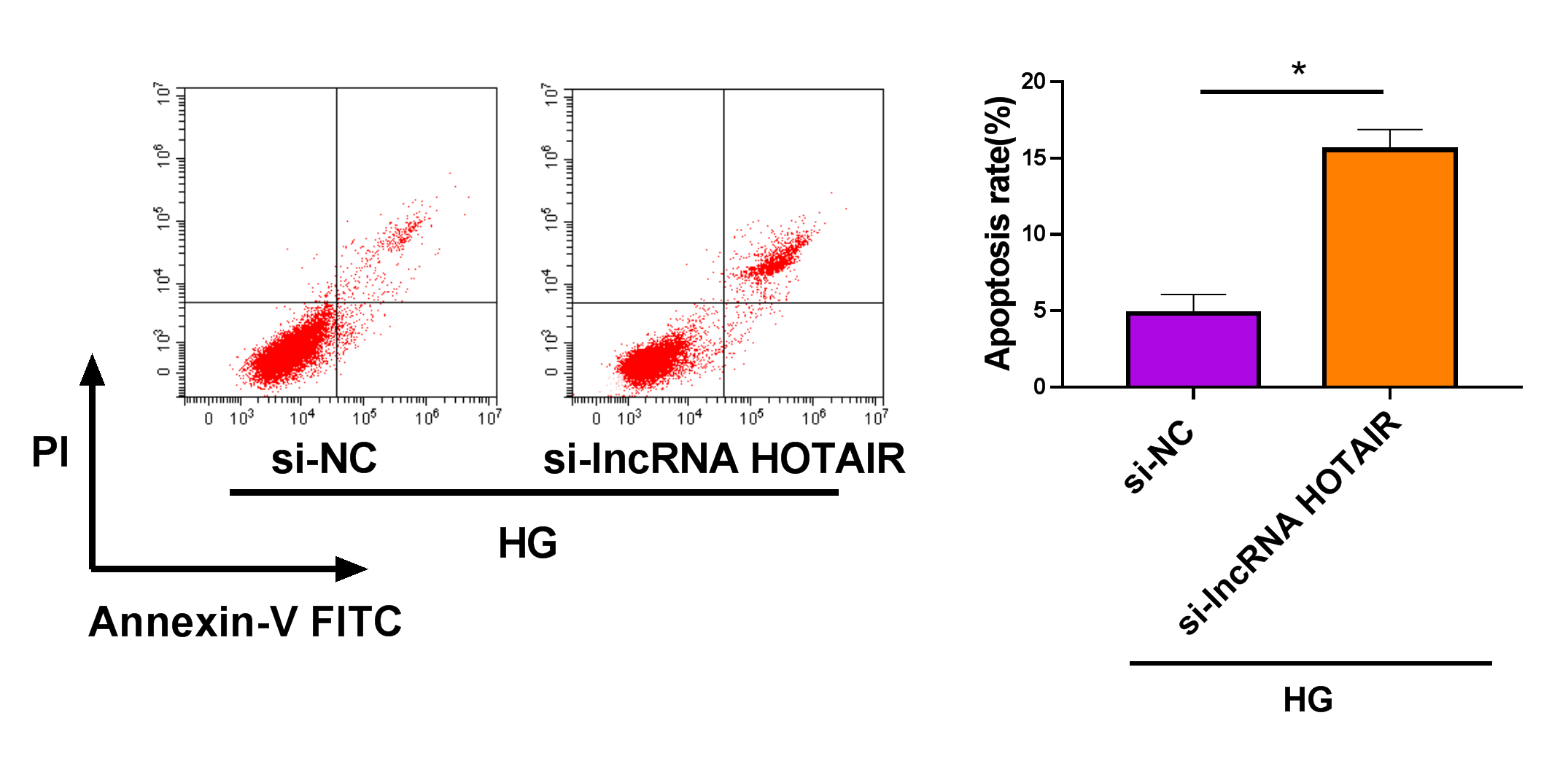

Supplement: Supplementary file 1 — Additional file 1: Figure S1. Effects of lncRNA HOTAIR silencing on the apoptosis of HG-induced HMC. HMC was transfected with si-NC or si-lncRNA HOTAIR, and then treated with HG. The apoptosis rate of HMC was assessed by flow cytometry. *P < 0.05. [file 13098_2022_802_MOESM1_ESM.tif]
